# Supplementary material for: Probiotic Sheep Milk: Physicochemical Properties of Fermented Milk and Viability of Bacteria Under Simulated Gastrointestinal Conditions
Source: Nutrients. 2025 Oct 24;17(21):3340. doi: 10.3390/nu17213340 (PMC12608772; doi:10.3390/nu17213340)
Supplement: Supplementary file 1 [file nutrients-17-03340-s001.zip › nutrients-3930254-supplementary.pdf]

Table S1. Pearson correlation coefficients.

pH7	0.45*														
L	-0.06	0.00													
a*	0.04	0.06	-0.68*												
b*	0.21	0.22	-0.72*	0.49*											
C*	0.22	0.30	-0.69*	0.45*	0.97*										
h^0	0.00	-0.01	0.70*	-0.98*	-0.47*	-0.41*									
TA%	-0.36*	-0.70*	-0.13	0.30	-0.03	-0.13	-0.31*								
Syneresis	-0.07	0.39*	-0.09	-0.33*	0.22	0.24	0.33*	-0.32*							
Consistency	-0.18	-0.25	0.50*	-0.64*	-0.38*	-0.31*	0.64*	-0.18	0.05						
Milky-Creamy Taste	-0.07	-0.13	0.32*	-0.18	-0.11	-0.03	0.21	0.01	-0.35*	0.43*					
Sour Taste	-0.17	0.03	0.16	-0.10	-0.22	-0.19	0.11	-0.01	-0.08	0.01	-0.02				
Sweet Taste	0.24	-0.13	-0.08	-0.06	0.14	0.09	0.04	-0.05	-0.11	0.03	0.18	-0.51*			
Off-Taste	-0.10	-0.20	-0.12	0.33*	-0.02	-0.06	-0.35*	0.51*	-0.24	-0.49*	-0.07	0.21	-0.21		
Sour Odor	-0.30	-0.09	0.27	0.03	-0.21	-0.19	-0.06	0.16	-0.08	0.17	0.02	0.50*	-0.45*	0.14	
Off-Odor	-0.01	-0.07	0.00	0.01	0.02	-0.08	-0.02	0.20	0.04	-0.34*	-0.29	0.23	0.12	0.52*	0.11
	Number of bacterial cells	pH 7	L	a*	b*	C*	h^0	TA%	Syneresis %	Consistency	Milky-Creamy Taste	Sour Taste	Sweet Taste	Off-Taste	Sour Odor

*- correlation coefficients significant at $p \leq 0.05$
